# Group I mGluR-Mediated Activation of Martinotti Cells Inhibits Local Cortical Circuitry in Human Cortex

**DOI:** 10.3389/fncel.2019.00315

**Published:** 2019-07-11

**Authors:** Tim Kroon, Julia Dawitz, Ioannis Kramvis, Jasper Anink, Joshua Obermayer, Matthijs B. Verhoog, René Wilbers, Natalia A. Goriounova, Sander Idema, Johannes C. Baayen, Eleonora Aronica, Huibert D. Mansvelder, Rhiannon M. Meredith

**Affiliations:** ^1^Department of Integrative Neurophysiology, Center for Neurogenomics and Cognitive Research, VU University Amsterdam, Amsterdam, Netherlands; ^2^Department of Neuropathology, Amsterdam Neuroscience, Amsterdam UMC, University of Amsterdam, Amsterdam, Netherlands; ^3^Stichting Epilepsie Instellingen Nederland, Heemstede, Netherlands; ^4^Department of Neurosurgery, VU University Medical Center, Amsterdam, Netherlands

**Keywords:** mGluR, human cortex, Martinotti, fast-spiking interneuron, LTD, single-cell RNA-sequencing

## Abstract

Group I metabotropic glutamate receptors (mGluRs) mediate a range of signaling and plasticity processes in the brain and are of growing importance as potential therapeutic targets in clinical trials for neuropsychiatric and neurodevelopmental disorders (NDDs). Fundamental knowledge regarding the functional effects of mGluRs upon pyramidal neurons and interneurons is derived largely from rodent brain, and their effects upon human neurons are predominantly untested. We therefore addressed how group I mGluRs affect microcircuits in human neocortex. We show that activation of group I mGluRs elicits action potential firing in Martinotti cells, which leads to increased synaptic inhibition onto neighboring neurons. Some other interneurons, including fast-spiking interneurons, are depolarized but do not fire action potentials in response to group I mGluR activation. Furthermore, we confirm the existence of group I mGluR-mediated depression of excitatory synapses in human pyramidal neurons. We propose that the strong increase in inhibition and depression of excitatory synapses onto layer 2/3 pyramidal neurons upon group I mGluR activation likely results in a shift in the balance between excitation and inhibition in the human cortical network.

## Introduction

Metabotropic glutamate receptors (mGluRs) form a diverse set of G-protein-coupled receptors that are divided into three groups, based on sequence homology, pharmacological properties, and signal transduction (Nakanishi, 1992). The most studied of the three is group I, which comprises mGluR1 and mGluR5, both of which act through Gq proteins. Group I mGluRs are located perisynaptically and are involved in a range of signaling and synaptic plasticity processes (Luján et al., 1996). They are particularly known for inducing a form of long-term depression (LTD) at glutamatergic synapses, which can be mediated by either mGluR1 or mGluR5, depending on brain region, postsynaptic cell type, and specific pathways in which the synapse is involved (Lüscher and Huber, 2010; Sherman, 2014). In addition to their role in LTD, group I mGluR activation potentiates NMDA-receptor-mediated currents (Wang and Daw, 1996; Mannaioni et al., 2001), and can depolarize several types of neurons through activation of a Ca^2+^-dependent cation conductance and decrease of resting K^+^ current (Baskys et al., 1990; Crepel et al., 1994; Guérineau et al., 1994, 1995).

While most studies of mGluR function, as well as its therapeutic effects, have centered upon excitatory signaling and pyramidal neurons (Chuang et al., 2000; Bandrowski et al., 2003), mGluRs can induce plasticity at GABAergic synapses through a variety of mechanisms (Galante and Diana, 2004; Valentinova and Mameli, 2016). Furthermore, group I mGluRs are expressed in several types of interneurons in both mouse and human brain (López-Bendito et al., 2002; Boer et al., 2010). Consequently, group I mGluRs depolarize specific types of interneurons (McBain et al., 1994; Van Hooft et al., 2000) and increase synaptic inhibition in rodent brain (Zhou and Hablitz, 1997; Mannaioni et al., 2001). Activation of group I mGluRs can also synchronize network activity by eliciting synchronous spiking in low-threshold spiking interneurons (Beierlein et al., 2000), which include Martinotti cells.

In recent years, group I mGluRs, and mGluR5 in particular, have become of increasing interest as potential therapeutic targets in neuropsychiatric and neurodevelopmental disorders (NDDs) (Barnes et al., 2015), including schizophrenia (Conn et al., 2009), and autistic spectrum disorders (ASDs) (Aguilar-Valles et al., 2015; Wenger et al., 2016). For example, dysregulated group I mGluR-mediated plasticity was proposed to underlie the NDD pathophysiology of fragile X syndrome (FXS) (Bear et al., 2004), since group I mGluR-mediated LTD is exaggerated in hippocampal pyramidal neurons in the FXS mouse model (Huber et al., 2002). Strikingly, mGluR-elicited spiking in Martinotti cells has been shown to be reduced in the Fmr1-KO mouse model for FXS (Paluszkiewicz et al., 2011b). These findings led to clinical trials targeting mGluR5 in adults with FXS (Berry-Kravis, 2014; Jacquemont et al., 2014). Unfortunately, these trials have thus far been unsuccessful, with reasons given ranging from patient age, and drug dosage level, to incomplete knowledge at a brain circuit rather than at a single cell level (Mullard, 2015; Berry-Kravis et al., 2016, 2018). Furthermore, rodent data on mGluR function has rarely been validated in the human brain. New work has started to confirm the existence of some of the effects of mGluRs in human cortex. The influence of group II mGluRs on glutamatergic transmission has recently been shown to be the same in human cortex as it is in rodents (Bocchio et al., 2019), as has mGluR-mediated LTD in fast-spiking interneurons (Szegedi et al., 2016). Given the importance of validation in humans of the basic mechanisms underlying therapies for cognitive disorders, we sought to confirm the effects of group I mGluRs in human cortex. Accordingly, we report that group I mGluRs increase inhibitory transmission onto several types of neurons in human cortex and identify depolarization of Martinotti cells as a potential mechanism. Furthermore, we confirm the existence of mGluR-mediated synaptic depression in human pyramidal neurons. Taken together, these results provide an essential step forward in understanding human mGluR-mediated signaling that may inform our understanding of their therapeutic actions in future clinical trials.

## Materials and Methods

### Acute Slice Preparation From Human Cortex

All procedures carried out involving patient tissue were approved by the VU University Medical Center Medical Ethical Committee and in accordance with the Dutch law and the declaration of Helsinki. All 40 patients provided written informed consent. The majority of cortical samples were taken from patients that suffered from drug-resistant epilepsy, in most cases due to hippocampal sclerosis (Table 1). During surgery, non-pathological tissue showing no structural abnormalities was resected from anterior and medial temporal cortex (Goriounova et al., 2018) (in this paper Figure 2 shows the exact location and extent of the resection and what tissue block was taken to the lab) in order to reach the pathological focus. Tissue was immediately stored and transported to the physiology laboratory in ice-cold slicing solution containing (in mM) 110 Choline chloride, 26 NaHCO3, 10 D-glucose, 11.6 sodium ascorbate, 7 MgCl2, 3.1 sodium pyruvate, 2.5 KCl, 1.25 NaH2PO4, and 0.5 CaCl2. 350–450 μm thick slices were prepared in the same, carbogenated, solution and were left to recover in aCSF containing (in mM) 125 NaCl, 26 NaHCO3, 10 D-glucose, 3 KCl, 2 CaCl2, 1 MgCl2, and 1.25 NaH2PO4 at 35°C, and then for at least 60 min at room temperature. aCSF in both recovery and recording chambers was continuously bubbled with a mixture of 95% O_2_ and 5% CO_2_.

**TABLE 1:** Patient data for all subjects used in this study.

**Patient**	**Age**	**Sex**	**Diagnosis**	**Brain region**	**Years of epilepsy**	**Seizure frequency**	**Medication used**
1	31	Female	MTS	Temporal	10	4/month	CBZ, CLB
2	25	Male	Tumour	Temporal	2	Absence seizures: 2/week; daily epigastric aura	LEV, CBZ, LCS
3	44	Female	MTS	Temporal	22	3/month	CBZ, CLB, LTG
4	47	Female	Tumour	Temporal	21	8/month	CBZ
5	38	Male	MTS	Temporal	10	6/month	CBZ
6	43	Male	MTS	Temporal	9	4/month	LCS, VPA
7	29	Female	MTS	Temporal	27.5	8.5/month	CLB, OXC
8	43	Male	MTS	Temporal	39.5	8/month	LTG, LEV
9	31	Female	MTS	Temporal	25.5	151/month	N/A
10	25	Male	Cavernoma	Temporal	10	8/month	LEV
11	56	Female	Hippocampal malrotation	Temporal	44	N/A	TPM, PHT, PGB, CBZ
12	35	Male	MTS	Temporal	12.5	1/4–6 weeks	CBZ, LEV
13	49	Male	MTS	Temporal	33	1/week to >1/day	CBZ
14	63	Female	Cavernoma	Temporal	12	1/week to >1/day	LTG, CLB, TPM
15	48	Female	MTS	Temporal	34	1–2/week to 3/day	ZNS, CBZ, VPA
16	40	Female	MTS	Temporal	24	1/month to >1/week	ZNS, LTG, CLB, MID
17	33	Female	MTS	Temporal	14	Up to 20/day	CBZ, LEV, CLB
18	52	Male	Unspecified epilepsy	Temporal	48	Up to 3/day; tonic-clonic: 1/month	CBZ, CLB
19	61	Male	MTS	Temporal	55	1/week	MID, LTG, PHB, PHT
20	51	Female	MTS	Temporal	32	40/month	CZP, LTG, OXC
21	21	Female	MTS	Temporal	N/A	N/A	N/A
22	57	Male	MTS	Temporal	7	4–5/month	LTG, OXC, ZNS
23	39	Male	MTS	Temporal	18	6/month	CBZ
24	17	Female	Tumour	Temporal	13	5/month	OXC
25	22	Male	Dysplasia	Occipital	12	8/month	VPA, OXC
26	47	Male	Dysplasia	Frontal	21	Variable; clustered	CBZ, LTG
27	41	Male	MTS	Temporal	40	1/month	CBZ, CLB, LEV
28	31	Female	MTS	Temporal	30	10/week	CBZ
29	60	Male	MTS	Temporal	14	1/month	LTG, LEV
30	24	Male	MTS	Temporal	7	3–5/month	CBZ, LCS, LEV
31	25	Female	Unspecified epilepsy	Temporal	14.5	Variable; clustered	CBZ, TPM
32	24	Female	MTS	Temporal	10.5	1–10/week	LTG, LEV
33	38	Female	Low grade lesion	Temporal	28	20/month	LTG
34	47	Female	MTS	Temporal	12.5	1–3/month	LEV
35	40	Male	Low grade lesion	Temporal	23	1-2/month	LEV, OXC
36	50	Female	MTS	Temporal	45	0–5/month	CBZ, PHT
37	51	Male	MTS	Temporal	49	Clusters: 7–8/year	LEV
38	32	Male	MTS	Temporal	8	25/month	CBZ, LCS, TPM, ZNS
39	38	Female	MTS	Temporal	32	4/day	CLB, LTG, LEV
40	44	Male	MTS	Temporal	N/A	3/week	VPA, OXC

*MTS, medial temporal sclerosis; CBZ, carbamazepine; CLB, clobazam; LCS, lacosamide; VPA, valproic acid; LTG, lamotrigine; OXC, oxcarbazepine; LEV, levetiracetam; TPM, topiramate; PGB, pregabalin; ZNS, zonisamide; MID, midazolam; PHB. Phenobarbital; PHT, phenytoin; CZP, clonazepam; ZNS, zonisamide; N/A, not available.*

### Electrophysiology

**FIGURE 1:**
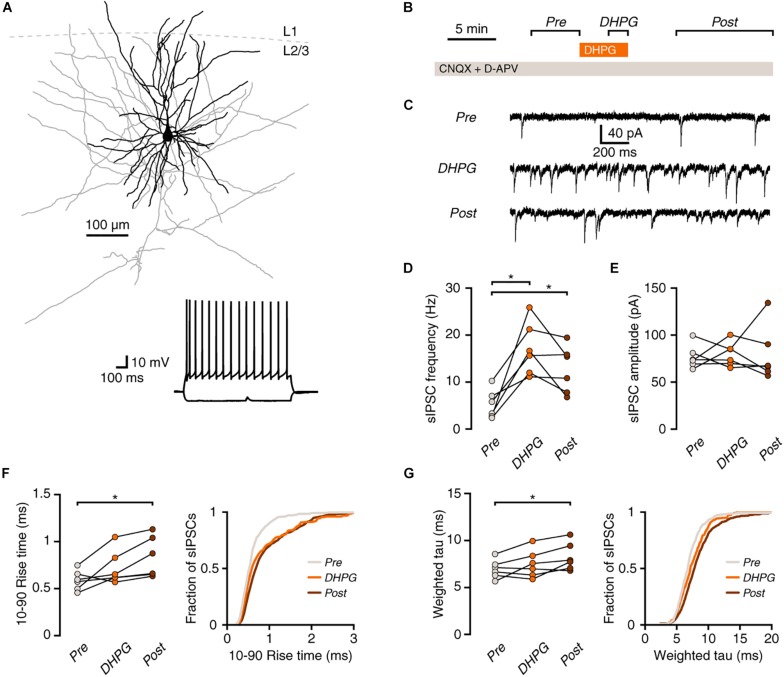
mGluR activation increases synaptic inhibition onto human pyramidal neurons. **(A)** Example morphological reconstruction of a human pyramidal neuron (350 μm slice; dendrites in black, axon in gray). Inset: electrophysiological response to negative and positive current steps. **(B)** Experimental protocol. **(C)** Example traces showing IPSCs before (Pre), during (DHPG) and after (Post) application of DHPG. **(D)** DHPG elicited a lasting increase in sIPSC frequency in pyramidal neurons (repeated-measures ANOVA: *F*(2,10) = 16.84, *p* = 0.003; Tukey’s *post hoc* test: Pre vs. DHPG ^*^p < 0.05, DHPG vs. Post ns, Pre vs. Post ^*^p < 0.05). **(E)** sIPSC amplitude was not significantly affected by DHPG [*F*(2,10) = 0.07, *p* = 0.929]. **(F)** Average rise time of sIPSCs in pyramidal neurons was slower after DHPG application [*F*(2,10) = 7.22, *p* = 0.011; Tukey’s *post hoc* test: Pre vs. DHPG ns, DHPG vs. Post ns, Pre vs. Post ^*^p < 0.05]. Right panel, cumulative probability distribution of sIPSC rise times, average of probability distributions calculated for each cell. **(G)** Decay time of sIPSCs was slower after DHPG application *F*(2,10) = 5.82, *p* = 0.021; Tukey’s *post hoc* test: Pre vs. DHPG ns, DHPG vs. Post ns, Pre vs. Post ^*^p < 0.05]. Right panel, cumulative probability distribution of sIPSC decay times, average of probability distributions calculated for each cell.

Slices in the recording chamber were perfused with aCSF heated to 31–33°C. Recordings were made using borosilicate (GC150-10, Harvard Apparatus, Holliston, MA, United States) glass pipettes with a resistance of 3 – 5 MΩ, pulled on a horizontal puller (P-87, Sutter Instrument Co., Novato, CA, United States). Signals were amplified (Multiclamp 700B, Molecular Devices), digitized (Digidata 1440A, Molecular Devices), and recorded in pCLAMP 10 (Molecular Devices, Sunnyvale, CA, United States). Access resistance was monitored before, during, and after recording. Cells were discarded if the access resistance deviated more than 25% from its value at the start of recording, or if it exceeded 20 MΩ. For current-clamp recordings and voltage-clamp recordings of excitatory postsynaptic current (EPSCs), pipettes contained intracellular solution consisting of (in mM) 148 K-gluconate, 1 KCl, 10 Hepes, 4 Mg-ATP, 4 K2-phosphocreatine, 0.4 GTP and 0.5% biocytin, adjusted with KOH to pH 7.3 (± 290 mOsm). All EPSC recordings except those shown in Figure 5H were performed in the presence of 10 μM Gabazine (Tocris Bioscience, Bristol, United Kingdom). To measure evoked EPSCs (eEPSCs), a pipette filled with aCSF was placed on a stimulation electrode and positioned within 100 μm from the recorded neuron. Current pulses were applied using an ISO-Flex stimulation box, and timed by a Master 9 (A.M.P.I., Jerusalem, Israel). The stimulation pipette was positioned so that a clear postsynaptic response could be observed with a clear separation from the stimulation artifact (Figure 1B). The stimulus intensity was set to evoke a half-maximal current. Pulses were applied every 15 s and a baseline of at least 5 min was recorded after the eEPSC amplitude stabilized. After recording a stable baseline, 25 μM DHPG was perfused into the recording chamber for 5 min. After a 5-min washout period, eEPSCs were measured every 15 s for up to 40 min and responses averaged per 10-min bins. In a subset of experiments, shown in Figure 5H, eEPSCs were recorded during DHPG application. These recordings were performed in the absence of GABAzine, so as not to elicit network events. Spontaneous inhibitory postsynaptic currents (sIPSCs) were measured using an intracellular solution containing (in mM) 70 K-gluconate, 70 KCl, 10 Hepes, 4 Mg-ATP, 4 K2-phosphocreatine, 0.4 GTP and 0.5% biocytin, adjusted with KOH to pH 7.3 (±290 mOsm). IPSC recordings were performed in the presence of 10 μM CNQX (Abcam, Cambridge, United Kingdom) and 50 μM D-APV (Abcam). sIPSCs were recorded from pyramidal neurons located in L2/3 and interneurons located in layer 1.

### *Post hoc* Morphological Assessment

Slices containing biocytin-filled cells were fixed in 4% paraformaldehyde in PBS for 24 – 48 h at 4°C. Slices were washed at least 3 × 10 min in PBS, and incubated in PBS containing 0.5% Triton X-100 and 1:500 Alexa 488-streptavidin (Invitrogen, Waltham, MA, United States) on a shaker at approximately 18–23°C (room temperature) for 48 h. Slices were then further washed at least 3 × 10 min in PBS and mounted on glass slides in mounting medium containing 0.1M Tris pH 8.5, 25% glycerol, 10% w/v Mowiol (Sigma-Aldrich). The morphology of recorded cells was checked for identification of their cell type (see Ascoli et al., 2008; Tremblay et al., 2016). Selected cells were imaged using an A1 confocal microscope (Nikon, Tokyo, Japan) using a 10×, NA 0.45 objective, scanned at 0.5 μm × 0.5 μm × 1.0 μm (xyz) resolution. Cellular morphology was reconstructed using NeuroMantic software (Myatt et al., 2012).

### Immunohistochemistry

To assess the expression of mGluR1α in somatostatin-positive neurons, temporal cortical tissue was used from patients undergoing surgery for mesial temporal lobe epilepsy (MTLE; 1 male, 2 female, 25 – 47 years) and three autopsy controls, displaying a normal cortical structure for the corresponding age and without any significant brain pathology (1 male, 2 female, 25 – 49 years). The control cases included in this study were selected from the databases of the Department of Neuropathology of the Academic Medical Center, University of Amsterdam, Amsterdam, Netherlands. Tissue was obtained during autopsy and used in accordance with the Declaration of Helsinki and the AMC Research Code provided by the Medical Ethics Committee. All autopsies were performed within 24 h after death. Tissue was fixed in 10% buffered formalin and embedded in paraffin. 6 μm sections were incubated overnight at 4°C in primary antibody solution (mGluR1α, 1:100, monoclonal mouse SC-55565, Santa Cruz Biotechnology, Santa Cruz, CA; Somatostatin, 1:300, polyclonal rabbit, AB1595, Chemicon, Temecula, CA, United States). Sections were then incubated for 2 h at room temperature with Alexa Fluor 568-conjugated anti-rabbit and Alexa Fluor 488 anti-mouse immunoglobulin G (IgG, 1:200, Thermo Fisher Scientific, Waltham, MA, United States). Finally, sections were analyzed using a laser scanning confocal microscope (Leica TCS Sp2, Wetzlar, Germany).

### Quantification of GRM1 and GRM5 Expression

GRM1 and GRM5 expression levels were quantified using publicly available Allen Institute for Brain Science (AIBS) database on human single-cell transcriptomics at http://celltypes.brain-map.org/, where the detailed methods can be found. The transcriptomic data from Allen Institute comes from human temporal cortical tissue, postmortem or surgically resected, sectioned and dissected per layer (Hodge et al., 2018). The methods include single nuclei fluorescence-activated cells sorting (FACS) isolation based on DAPI and neuronal nuclei staining (NeuN), followed by Smart-seq v4 based library preparation and single-cell deep (2.5 million reads/cell) RNA-Seq.

The data on single nucleus GRM1 and GRM5 mRNA expression in transcriptomic types from AIBS database were pooled to represent higher-order hierarchical clusters (SST, PVALB, PAX6/LAMP5, and excitatory types) from selected cortical layers of interest. Violin plots were made using custom-made Matlab scripts (Mathworks, Natick, MA, United States), the plots represent distribution of mRNA expression on a log scale with counts per million (CPM) value of 4000.

### Analysis and Statistics

Electrophysiological data were analyzed using custom scripts in Matlab. All data are represented as mean ± standard error of the mean (SEM). Normal distribution of the data was tested using Shapiro-Wilk tests. Appropriate statistical tests were performed in Prism 7 (Graphpad, La Jolla, CA, United States), and are mentioned in the figure legends.

## Results

### Group I mGluR Activation Increases Inhibition Onto Human Pyramidal Neurons

Activation of group I mGluRs increases spontaneous inhibition in rodent cortex (Paluszkiewicz et al., 2011b). To test whether this holds true in human cortex, we recorded spontaneous inhibitory postsynaptic currents (sIPSCs) in pyramidal neurons in layer 2/3 of surgically resected human neocortex and activated group I mGluRs by a 5-min bath application of the agonist (S)-3,5-Dihydroxyphenylglycine (DHPG; Figures 1A–C). Application of DHPG led to an increase in the frequency of sIPSCs in pyramidal neurons that lasted after the agonist washout from the bath (Figure 1D). Interestingly, while the amplitude of inhibitory events was unaffected (Figure 1E), both the rise and decay times were increased after washout of the agonist (Figures 1F,G).

### Group I mGluRs Strongly Activate Martinotti Cells in Human Cortex

**FIGURE 2:**
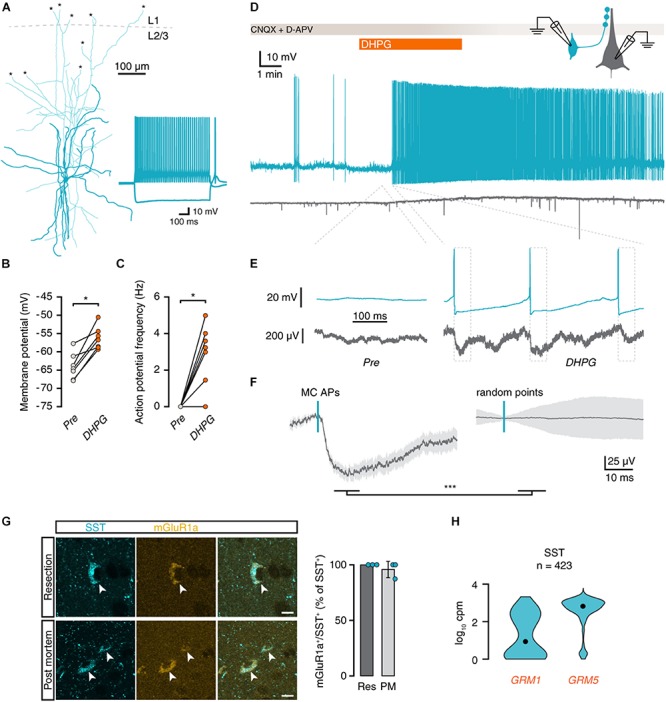
mGluR activation depolarizes Martinotti cells and leads to action potential firing. **(A)** Morphological reconstruction of an MC in human cortex (350 μm slice). Morphology was recovered *post hoc* for 5 out of 7 recorded cells. Asterisks denote places where a neurite was cut during slice preparation. Inset: electrophysiological response to negative and positive current steps. **(B)** Membrane potentials of MCs are depolarized by DHPG (Wilcoxon matched-pairs signed rank test, *n* = 7, W = 28, *p* = 0.016). **(C)** DHPG induced an increase in action potential frequency (Wilcoxon matched-pairs signed rank test, *n* = 7, W = 21, *p* = 0.031). **(D)** Voltage traces of a connected pair consisting of an MC (teal) and a pyramidal neuron (gray). Application of DHPG (orange) induces sustained action potential spiking in the MC. **(E)** Voltage traces of MC and pyramidal neuron before and during application of DHPG (dashed lines indicate corresponding area of the trace in **D**). Dashed boxes denote the area used for the analysis in f. **(F)** Average pyramidal neuron voltage trace (left panel, 50 events, light gray area shows SEM) around MC action potentials (left panel, teal dash) shows an inhibitory response that is absent in voltage traces centered on random time points during the same period (right panel; Mann-Whitney *U* = 418, *p* < 0.001). **(G)** Immunohistochemical staining for somatostatin (cyan) and mGluR1a (yellow) shows that mGluR1a is present in SST^+^ interneurons (arrowheads) in both resected and post-mortem tissue. Scale bar = 10 μm. Right panel: percentage of SST^+^ cells positive for mGluR1a per subject. **(H)** Distribution of *GRM1* and *GRM5* RNA levels in SST^+^ cells. Data taken from the Allen Institute human single-cell RNA-seq database. Here and further, black dot shows the median, n number above is the number of cells (nuclei) plotted.

A potential cause of the slower kinetics would be a change in membrane time constant caused by DHPG. However, the membrane time constant after completion of the experiment did not differ from that measured before the start of the experiment [before: 17.7 ± 2.2 ms, after: 15.3 ± 3.0 ms, paired *t*(5) = 0.931, *p* = 0.395]. As inputs that are further away from the soma appear to have slower kinetics due to the filtering properties of dendrites (Magee, 2000), we hypothesized that the slower synaptic inputs elicited by DHPG might be onto distal dendrites and were therefore likely coming from Martinotti cells (MCs). We performed current-clamp recordings of putative MCs in layer 2/3 to assess whether group I mGluR activation would elicit a change in membrane potential. Putative MCs were identified by an ovoid-shaped cell body and bitufted proximal dendritic morphology in the DIC microscopic image and by a rebound action potential following a depolarizing current step. *Post hoc* reconstruction of the morphology of these cells showed that the axon of putative MCs branched out and terminated in layer 1 (Figure 2A; Obermayer et al., 2018). Application of DHPG caused a depolarization of 7.7 ± 1.4 mV before the start of action potential firing (Figure 2B) and led to action potential firing in 6 out of 7 MCs (Figure 2C). In one experiment, a connected pair of MC and pyramidal neuron was recorded (Figure 2D). Upon DHPG application, the MC started firing action potentials, and the pyramidal neuron received an increased number of inhibitory postsynaptic potentials (IPSPs, Figure 2E). Analysis of the pyramidal neuron membrane potential following 50 MC action potentials showed distinct IPSPs (Figure 2F, left panel). Performing the same analysis on randomly generated time points did not show a similar peak (Figure 2F, right panel; *p* < 0.001). The latency between the peak of the MC action potential and the onset of IPSPs in the pyramidal neuron was 1.75 ms, with a jitter of 396 μs. Thus, action potentials elicited by DHPG in the presynaptic MC generate time-locked inhibitory responses in postsynaptic pyramidal neurons.

To confirm that DHPG could mediate its effect on local synaptic inhibition directly via Martinotti cells, we performed double-labeling immunohistochemistry for somatostatin and mGluR1a. We observed near-total colocalization of mGluR1a and somatostatin in samples from both surgically resected (22 out of 22 SST+ neurons from 3 samples) and *post mortem* (22 out of 23 SST+ neurons from 3 samples) human temporal cortex (Figure 2G). In addition, single-cell RNA-sequencing data from the Allen Brain Institute showed strong expression of both *GRM1* and *GRM5* in human SST^+^ interneurons (Figure 2H). We therefore conclude that Martinotti cells are equipped with group I mGluRs to directly respond to DHPG and mediate the increase in synaptic inhibition observed in pyramidal neurons in superficial layers of human temporal cortex following group I mGluR activation.

### Synaptic Inhibition Onto Layer 1 Interneurons Is Increased by Group I mGluR Activation

**FIGURE 3:**
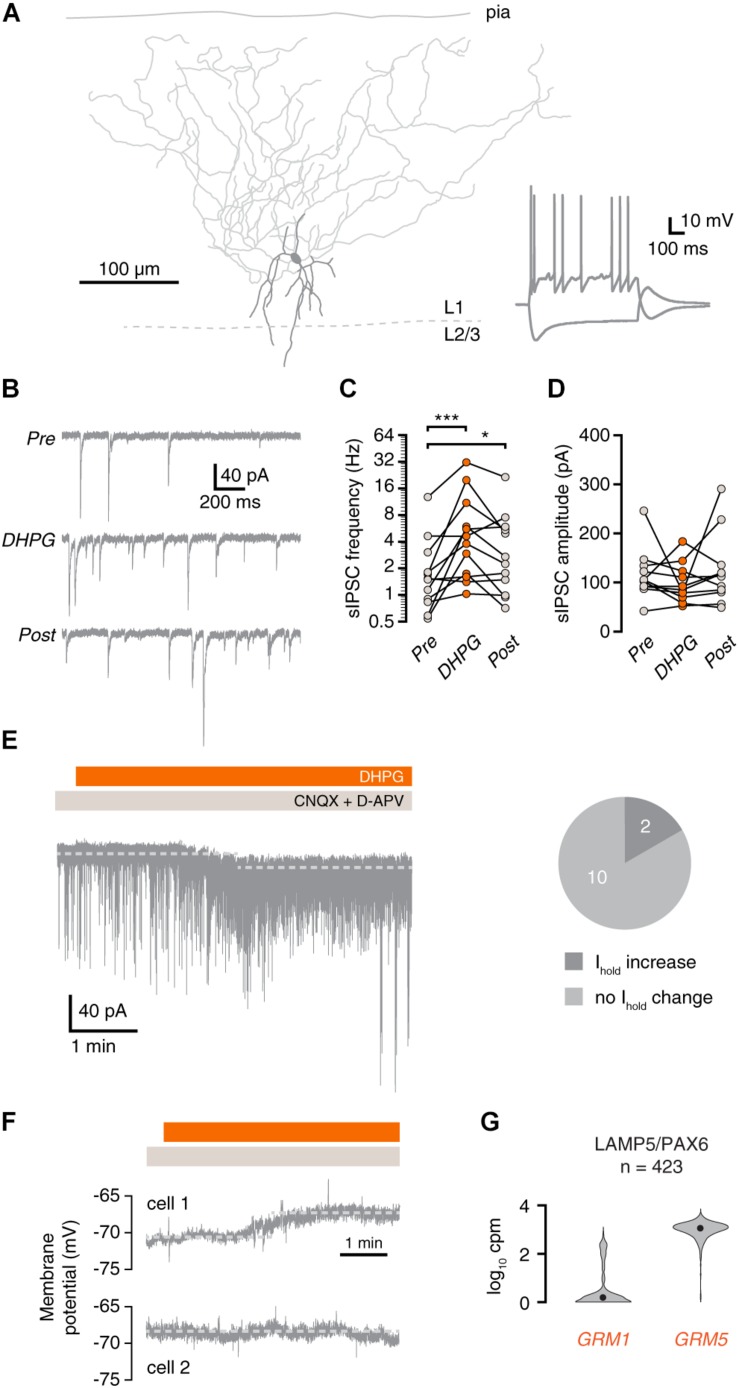
mGluR activation increases synaptic inhibition onto layer 1 interneurons. **(A)** Morphological reconstruction of a human L1 interneuron (350 μm slice). Morphology was recovered *post hoc* for 11 out of 15 recorded cells. Inset: electrophysiological response to negative and positive current steps. **(B)** Example traces showing IPSCs before (Pre), during (DHPG) and after (Post) application of DHPG. **(C)** DHPG elicited a prolonged increase in sIPSC frequency in L1 interneurons (repeated-measures ANOVA; *F*(2,22) = 12.09, *p* < 0.001; Tukey’s *post hoc* test: Pre vs. DHPG ^∗∗∗^*p* < 0.001, DHPG vs. Post ns, Pre vs. Post ^*^p < 0.05]. **(D)** sIPSC amplitude in L1 interneurons was not significantly affected by DHPG [repeated-measures ANOVA; *F*(2,20) = 1.16, *p* = 0.333]. **(E)** Example current trace showing increased sIPSC frequency and shift in holding current upon DHPG bath application. Right panel, proportion of cells in which the holding current shifted upon DHPG application. **(F)** L1 interneurons are depolarized (cell 1, upper panel) or were unresponsive (cell 2, lower panel) to DHPG application. **(G)** *GRM1* and *GRM5* RNA levels in L1 interneurons. Data taken from the Allen Institute human single-cell RNA-seq database.

Martinotti cells are known to contact most types of interneurons in addition to pyramidal neurons. Therefore, we tested whether interneurons in layer 1 (L1) of the human cortex also receive more inhibitory input upon group I mGluR activation. To this end, we recorded sIPSCs in L1 interneurons (Figures 3A,B). Similar to pyramidal neurons, sIPSC frequency onto L1 interneurons was increased during and after application of DHPG (Figure 3C), without a change in sIPSC amplitude (Figure 3D). In addition to increased sIPSC frequency, 2 out of 12 L1 interneurons showed a small increase in holding current after DHPG application (Figure 3E). This increase in holding current corresponds to a depolarization of 5.4 and 6.7 mV when taking into account the input resistance of the cells. DHPG-induced depolarization in L1 interneurons is therefore unlikely to elicit action potentials. During current-clamp recordings, L1 interneurons exhibited a small depolarization or no response, but did not fire action potentials in response to DHPG (Figure 3F, *n* = 3). Thus, we did not find any evidence that L1 interneurons contribute to the increase in synaptic inhibition upon group I mGluR activation. In accordance with this, human L1 interneurons express *GRM5*, but only rarely express *GRM1* according to Allen Brain Institute single-cell sequencing data (Figure 3G).

### Group I mGluRs Depolarize Fast-Spiking Interneurons, but Do Not Elicit Action Potential Firing

**FIGURE 4:**
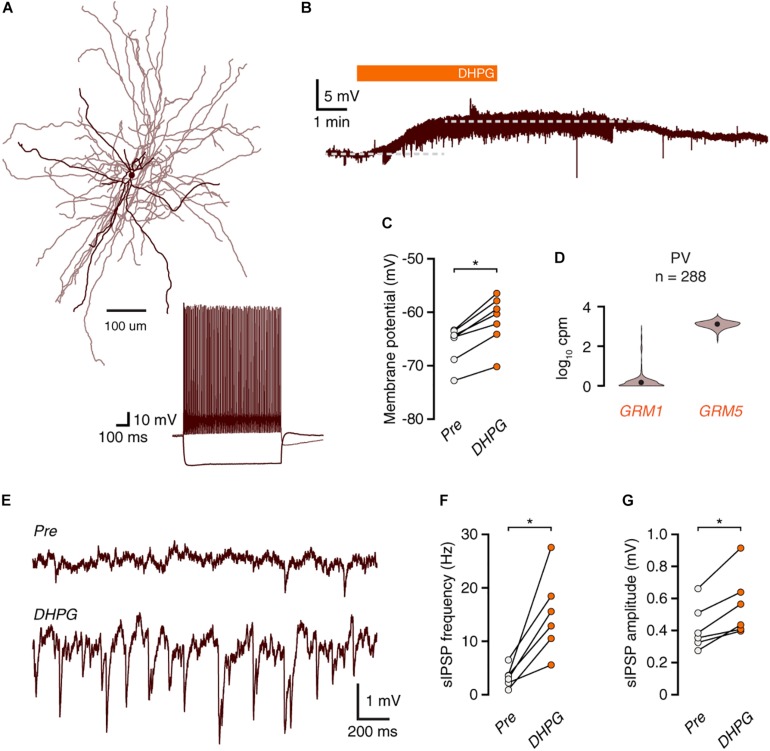
mGluR activation depolarizes FS interneurons without leading to action potential firing. **(A)** Morphological reconstruction of a human fast-spiking basket cell (350 μm slice). Morphology was recovered *post hoc* for 4 out of 7 recorded cells. Inset: electrophysiological response to negative and positive current steps. **(B)** Voltage trace showing depolarization of a fast-spiking interneuron in response to DHPG. **(C)** Fast-spiking interneurons are depolarized by DHPG (Wilcoxon matched-pairs signed rank test, *n* = 7, W = –28, ^*^p = 0.016). **(D)** *GRM1* and *GRM5* RNA levels in FS parvalbumin^+^ interneurons. Data taken from the Allen Institute human single-cell RNA-seq database. **(E)** Representative traces showing an increase in inhibitory synaptic potentials during DHPG bath application compared to baseline. **(F)** DHPG increased the frequency of spontaneous inhibitory events in fast-spiking interneurons (Wilcoxon matched-pairs signed rank test, *n* = 6, W = 21, *p* = 0.031). **(G)** sIPSP amplitudes are increased by DHPG application (Wilcoxon matched-pairs signed rank test, *n* = 6, W = 21, *p* = 0.031).

In rodents, fast-spiking (FS) interneurons can be depolarized by activation of group I mGluRs. To assess whether FS interneurons contribute to DHPG-induced inhibition in human cortex, we performed current-clamp recordings of FS interneurons (Figures 4A,B). Application of DHPG led to depolarization of all recorded FS interneurons (Figure 4C, *n* = 7), but did not elicit action potential firing. In accordance with these results, analysis of single-cell sequencing data revealed that, similar to L1 interneurons, human PV^+^ FS interneurons express *GRM5*, rather than *GRM1* (Figure 4D). DHPG application did lead to an increase in the frequency and amplitude of IPSPs (Figures 4E–G). Although this increase in IPSP frequency is likely due to increased MC activity, it could also be caused by an increase in driving force due to the depolarized membrane potential, which would facilitate detection of events. However, we found no significant correlation between the increase in IPSP frequency and the level of membrane depolarization among FS interneurons (Spearman’s *R* = −0.26, *p* = 0.62). Thus, FS interneurons receive increased synaptic inhibition upon group I mGluR activation, but are themselves not likely to contribute to this effect.

### Excitatory Inputs Onto Human Pyramidal Neurons Exhibit mGluR-Mediated Depression

**FIGURE 5:**
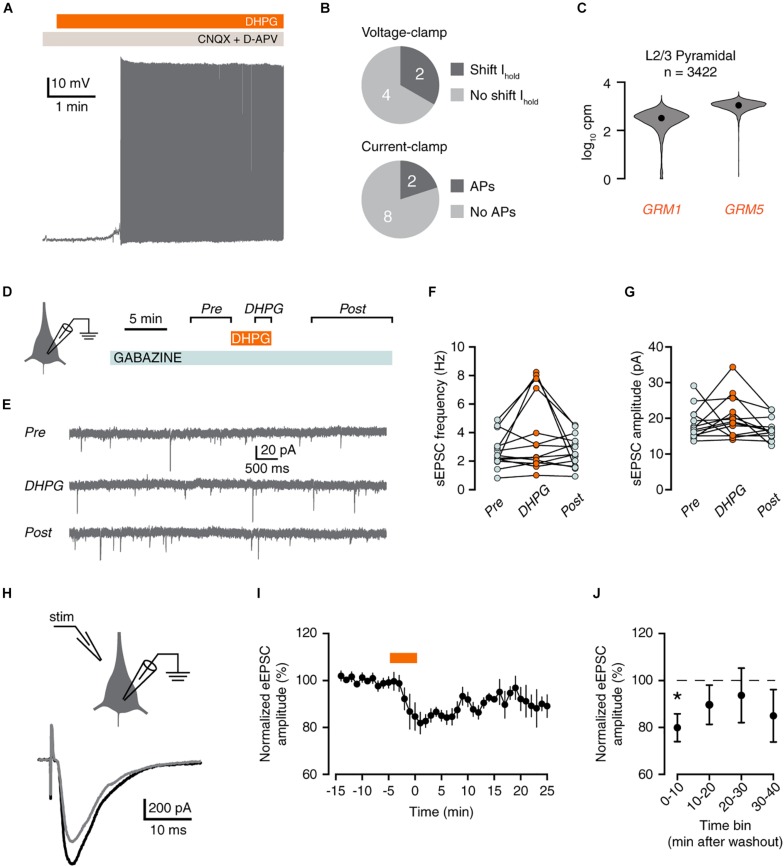
mGluR activation reduces excitatory inputs to pyramidal neurons. **(A)** Voltage trace showing DHPG-induced action potential firing in a pyramidal neuron. **(B)** Proportion of pyramidal neurons that displayed a shift in holding potential in voltage-clamp (top panel, neurons from Figure 1). Lower panel, proportion of pyramidal neurons that fired action potentials in response to DHPG in current-clamp. **(C)** *GRM1* and *GRM5* RNA levels in L2/3 pyramidal neurons. Data taken from the Allen Institute human single-cell RNA-seq database. **(D)** Experimental protocol for recording sEPSCs. **(E)** Example current traces showing sEPSCs. **(F)** sEPSC frequency was not increased by DHPG [Friedman test, χ^2^(2) = 3, *p* = 0.223]. **(G)** sEPSC frequency did not change significantly upon DHPG application [repeated-measures ANOVA: *F*(2,24) = 2.55, *p* = 0.122]. **(H)** Experimental protocol for recording evoked EPSCs, depicting placement of stimulus pipette (left panel), and example evoked responses (right panel) before (black) and after (gray) DHPG application. **(I)** Example of eEPSC responses during wash-in of DHPG (orange bar). Mean ± SEM of 4 responses binned per min. **(J)** DHPG decreased eEPSC amplitude up to 10 min after wash-out of DHPG [Friedman test, χ^2^(4) = 11.8, *p* = 0.019. *Post hoc*: Bonferroni-corrected Wilcoxon matched-pairs signed rank test, 10 min vs. baseline, *n* = 8, ^*^p < 0.05].

Finally, we examined whether excitatory inputs were equally affected by group I mGluR activation. In current-clamp, only 2 out of 10 pyramidal neurons responded to DHPG by firing action potentials (Figures 5A,B), although most L2/3 pyramidal neurons express *GRM1 and GRM5* (Figure 5C).

We therefore examined whether DHPG increased excitatory inputs onto pyramidal cells by measuring spontaneous excitatory postsynaptic currents (sEPSCs; Figures 5D,E). Application of DHPG transiently increased sEPSCs by 25% or more in 6 out of 14 pyramidal neurons. However, there was no significant increase in sESPC frequency overall (Figure 5F).

Group I mGluRs are known to induce depression of excitatory synapses. This is mediated by mGluR5, which virtually all L2/3 pyramidal neurons express (Figure 5C). To test whether human pyramidal neuron excitatory synapses undergo mGluR-mediated depression, we evoked EPSCs (eEPSCs) by electrical stimulation (Figure 5G). Indeed, application of DHPG acutely decreased the amplitude of eEPSCs relative to baseline (Figures 5H–J). Therefore, we conclude that pyramidal neurons in human cortex exhibit group I mGluR-mediated depression of excitatory synapses.

## Discussion

In this study, we addressed how activation of group I mGluRs affects microcircuits in superficial layers of the human neocortex. Our data demonstrate a cell-type specific recruitment of human cortical interneurons by group I mGluR activation. We find that Martinotti cells are strongly excited by group I mGluR activation, which increases the amount of inhibitory inputs to neighboring L2/3 pyramidal neurons. Somatostatin-positive interneurons in superficial layers of the human neocortex show strong abundance of mRNA for mGluR1 and mGluR5 receptors. Other local interneuron types, including fast spiking interneurons and layer I interneurons are depolarized by group I mGluR activation, but do not fire action potentials in response to this depolarization. Also, these interneuron types show a lower abundance of *GRM1* and *GRM5* mRNA. Furthermore, excitatory inputs to pyramidal neurons are suppressed by group I mGluR activation. Thus, the large increase in synaptic inhibition across cell types in superficial cortical layers and the depression of excitatory synapses most likely results in a net shift in the balance between excitation and inhibition in the cortical network.

In rodents, layer I interneurons and deep layer fast-spiking interneurons have previously been reported to fire action potentials upon mGluR activation with quisqualic acid (Zhou and Hablitz, 1997). We did not observe induced action potential firing in any human layer I interneuron or fast-spiking interneuron. This discrepancy could be due to the difference in pharmacological ligands used in the earlier study, which also activate ionotropic glutamate receptors in addition to metabotropic receptors. Our data are in agreement with metabotropic-specific ligand effects upon fast-spiking interneurons (Beierlein et al., 2000) and layer 1 cortical interneurons in rodents (Cosgrove and Maccaferri, 2012). Enhanced synaptic inhibition in fast-spiking interneurons and in layer 1 Cajal-Retzius cells is mediated by Martinotti cells in rodents. This effect is mediated by mGluR1a specifically (Beierlein et al., 2000; Cosgrove and Maccaferri, 2012). Therefore, we propose that Martinotti cells mediate enhanced synaptic inhibition in human superficial temporal cortex in response to group I mGluR activation. While we did not see direct action potential firing in any other interneuron types besides putative Martinotti cells, we cannot exclude the possibility that other interneuron types may also be involved in the mGluR-mediated increase in synaptic inhibition we observed.

Our results show that activation of group I mGluRs can directly depolarize both Martinotti cells and fast-spiking interneurons. Since group I mGluRs are located mostly perisynaptically and can therefore likely be activated by spillover of glutamate from the synaptic cleft (Luján et al., 1996), subsequent depolarization of these interneuron types may constitute a mechanism by which inhibition is increased upon a prolonged or very strong initial excitatory drive. Group I mGluR activation can alter neuronal excitability through a variety of differing mechanisms, including protein kinase C-mediated changes upon ion channels, or through calcium-dependent modulation of ion channels (Correa et al., 2017). mGluRs have been proposed to be involved in epileptogenesis (McNamara et al., 2006) and group I mGluRs are upregulated in the hippocampus of patients with temporal lobe epilepsy (Blümcke et al., 2000). In addition, studies have shown that the activation of mGluRs in hippocampal slices can increase epileptiform activity (Merlin and Wong, 1997). However, these studies often block GABAergic signaling in order to induce epileptiform activity, thereby disregarding the strong effect on inhibition we show here, and that is also observed in rodent hippocampus (McBain et al., 1994; Van Hooft et al., 2000). We therefore speculate that increased expression of mGluRs in epilepsy patients could be a homeostatic mechanism, rather than a direct component of the pathophysiology of epileptogenesis. In both cortex and hippocampus, group I mGluR-mediated increase in the frequency of inhibitory events is mediated by mGluR1 (Mannaioni et al., 2001; Sun and Neugebauer, 2011; Cosgrove and Maccaferri, 2012). We observed consistent co-expression of mGluR1a and somatostatin in putative Martinotti cells from both surgically resected tissue and autopsy controls. However, because group I mGluRs have different roles in different populations of neurons (Mannaioni et al., 2001; Volk et al., 2006), it remains to be determined whether mGluR1 or mGluR5 is solely responsible for the functional effects demonstrated here. Specifically, we found that FS and a subset of L1 interneurons are depolarized to some extent by DHPG, an effect that might be due to activation of mGluR5, which both types express.

We found group I mGluR-mediated depression of excitatory synapses received by L2/3 pyramidal neurons, similar to that observed in the rodent brain. Group I mGluR-LTD has previously been shown in human cortex for excitatory synapses onto fast-spiking interneurons (Szegedi et al., 2016). The finding of LTD at excitatory synapses on pyramidal neurons is similar to that in rodent hippocampus (Huber et al., 2000). The LTD we observed is not particularly strong and is shorter in duration than has been found previously (Huber et al., 2000). It is worth mentioning that while other studies in rodents typically use 100 μM DHPG, we only used 25 μM due to its strong acute excitation of network activity. Since the efficacy of DHPG in inducing LTD is dose-dependent (Ayala et al., 2009), this may explain why the LTD we observed was relatively small and short-lived. Overall, however, we demonstrate the occurrence of group I mGluR-induced LTD as a plasticity mechanism conserved across species, which means the aberrant LTD underlying the mGluR theory of FXS (Bear et al., 2004) may also apply to mature human cortex. However, to test mGluR-mediated LTD in FXS patient brain tissue would require using postmortem brain tissue for neurophysiological recordings (Kramvis et al., 2018), since surgically resected tissue as used in this study is not available from FXS patients.

In contrast to evoked excitatory responses, mean amplitudes of spontaneous events were not decreased by mGluR activation in our experiments. Group I mGluRs have been shown to increase the amplitude of excitatory synaptic spontaneous events in rodent somatosensory cortex (Bandrowski et al., 2003) and in rodent hippocampal interneurons (McBain et al., 1994). It is possible that in our recordings, mGluR-induced depression of a subpopulation of synapses is masked by a simultaneous global increase in events of a relatively large amplitude (Bandrowski et al., 2003), and that synaptic depression is visible only during the simultaneous timed activation of multiple synapses that occurs when synaptic events are evoked using extracellular stimulation. Conversely, a depression of excitatory synapses might cause the smaller responses from these synapses to fall below the detection threshold for spontaneous events. This might also explain why we observed no increase in the frequency of sEPSCs in most pyramidal neurons, even though the increase in action potential firing in a subset of pyramidal neurons is quite robust, and we find an increase in sESPC frequency in superficial interneurons. That only a subset of pyramidal neurons responded to mGluR activation may indicate that there are functional subtypes of pyramidal neurons in superficial human cortex that could be distinguished by differential mGluR expression. Indeed, superficial human pyramidal neurons can be divided into two classes based on morphology and electrotonic properties and their somatic location within the cortex corresponds to specific ion channel expression (Deitcher et al., 2017; Kalmbach et al., 2018). It remains to be determined whether these subtypes correspond to pyramidal neurons that do or do not respond to mGluR activation, or whether mGluR responsiveness further subdivides one or both of these classes.

Finally, recent studies using human cortical tissue have shown that there are fundamental differences in how rodent and human neurons function (Verhoog et al., 2013; Testa-Silva et al., 2014; Mohan et al., 2015; Wang et al., 2015; Eyal et al., 2016; Beaulieu-Laroche et al., 2018; Kalmbach et al., 2018). It should be noted that although the human neocortex used shows no structural abnormalities, patients typically had a long history of seizures and had been exposed to a variety of anti-epileptic medications, thus we cannot conclude unequivocally that these factors have not influenced neuronal function in some form. However, specific cholinergic mechanisms and modulation of disynaptic inhibition between cortical pyramidal neurons are conserved between rodents and humans (Obermayer et al., 2018; Poorthuis et al., 2018), as are the action of group II mGluRs (Bocchio et al., 2019), and group I mGluR-dependent LTD of excitatory synapses onto fast-spiking interneurons (Szegedi et al., 2016). We show here that several aspects of group I mGluR activation in the cortex are preserved across these mammalian species. The balance of synaptic excitation to inhibition and the role for aberrant mGluR signaling is of increasing focus for the synaptic, network and behavioral phenotypes related to rodent NDD and neuropsychiatry models (Levenga et al., 2010; Barnes et al., 2015; Nelson and Valakh, 2015; Lee et al., 2017). Notably, the specific aspects of group I mGluR function we validate as occurring in human cortex are also dysregulated in mouse models for FXS, notably enhanced LTD in hippocampal pyramidal neurons (Bear et al., 2004) and altered GABAergic inhibitory function specifically mediated by mGluR1 (Paluszkiewicz et al., 2011a; Cea-Del Rio and Huntsman, 2014). At the start of the 21st century, just over one third of all licensed and approved pharmaceutical drugs directly or indirectly modulated G-protein coupled receptors (Klabunde and Hessler, 2002). However, our fundamental knowledge on the function of G-protein coupled receptors, specifically mGluRs, and their specificity of action upon different neuronal subtypes within the human brain is far from complete. Therefore, we believe that our data have direct implications for interpreting the actions of group I mGluR-mediated signaling not only in human cortical circuits, but for translational approaches when designing clinical models from NDD rodent data to test specific mGluR targets therapeutically.

## Data Availability

The raw data supporting the conclusions of this manuscript will be made available by the authors, without undue reservation, to any qualified researcher.

## Ethics Statement

This study was carried out in accordance with the recommendations of the VU University Medical Center Medical Ethical Committee and in accordance with the Dutch law and the declaration of Helsinki. All 40 patients provided written informed consent.

## Author Contributions

TK, RM, JD, IK, and HM designed the study. TK, JD, IK, JA, JO, MV, and RW performed the experiments. TK, JD, IK, NG, and EA analyzed the data. SI and JB performed neurosurgery. TK, RM, and HM wrote the manuscript.

## Conflict of Interest Statement

The authors declare that the research was conducted in the absence of any commercial or financial relationships that could be construed as a potential conflict of interest.
